# Epidemiology and Prognostic Importance of Atrial Fibrillation in Kidney Transplant Recipients: A Meta-Analysis

**DOI:** 10.3390/jcm7100370

**Published:** 2018-10-19

**Authors:** Charat Thongprayoon, Ronpichai Chokesuwattanaskul, Tarun Bathini, Nadeen J. Khoury, Konika Sharma, Patompong Ungprasert, Narut Prasitlumkum, Narothama Reddy Aeddula, Kanramon Watthanasuntorn, Sohail Abdul Salim, Wisit Kaewput, Felicitas L. Koller, Wisit Cheungpasitporn

**Affiliations:** 1 Division of Nephrology and Hypertension, Mayo Clinic, Rochester, MN 55905, USA; charat.thongprayoon@gmail.com; 2 Division of Cardiovascular Medicine, Faculty of Medicine, Chulalongkorn University and King Chulalongkorn Memorial Hospital, Thai Red Cross Society, Bangkok 10330, Thailand; drronpichaic@gmail.com; 3 Department of Internal Medicine, University of Arizona, Tucson, AZ 85721, USA; tarunjacobb@gmail.com; 4 Division of Nephrology and Hypertension, Henry Ford Health System, Detroit, MI 48202, USA; nadeenj.khoury@gmail.com; 5 Department of Internal Medicine, Bassett Medical Center, Cooperstown, NY 13326, USA; drkonika@gmail.com (K.S.); kanramon@gmail.com (K.W.); 6 Clinical Epidemiology Unit, Department of Research and Development, Faculty of Medicine, Siriraj Hospital, Mahidol University, Bangkok 10700, Thailand; p.ungprasert@gmail.com; 7 Department of Internal Medicine, University of Hawaii, Honolulu, HI 96822, USA; narutprasitlumkum@gmail.com; 8 Division of Nephrology, Department of Medicine, Deaconess Health System, Evansville, IN 47747, USA; dr.anreddy@gmail.com; 9 Division of Nephrology, Department of Medicine, University of Mississippi Medical Center, Jackson, MS 39216, USA; sohail3553@gmail.com; 10 Department of Military and Community Medicine, Phramongkutklao College of Medicine, Bangkok 10400, Thailand; wisitnephro@gmail.com; 11 Department of Transplant and Hepatobiliary Surgery, University of Mississippi Medical Center, Jackson, MS 39216, USA; fkoller@umc.edu

**Keywords:** atrial fibrillation, kidney transplantation, renal transplantation, transplantation, systematic reviews, meta-analysis

## Abstract

This meta-analysis was conducted with the aims to summarize all available evidence on (1) prevalence of pre-existing atrial fibrillation (AF) and/or incidence of AF following kidney transplantation; (2) the outcomes of kidney transplant recipients with AF; and (3) the trends of estimated incidence of AF following kidney transplantation over time. A literature search was conducted utilizing MEDLINE, EMBASE, and the Cochrane Database from inception through March 2018. We included studies that reported (1) prevalence of pre-existing AF or incidence of AF following kidney transplantation or (2) outcomes of kidney transplant recipients with AF. Effect estimates from the individual study were extracted and combined utilizing random-effect, generic inverse variance method of DerSimonian and Laird. The protocol for this meta-analysis is registered with PROSPERO (International Prospective Register of Systematic Reviews; no. CRD42018086192). Eight cohort studies with 137,709 kidney transplant recipients were enrolled. Overall, the pooled estimated prevalence of pre-existing AF in patients undergoing kidney transplantation was 7.0% (95% CI: 5.6–8.8%) and pooled estimated incidence of AF following kidney transplantation was 4.9% (95% CI: 1.7–13.0%). Meta-regression analyses were performed and showed no significant correlations between year of study and either prevalence of pre-existing AF (*p* = 0.93) or post-operative AF after kidney transplantation (*p* = 0.16). The pooled odds ratios (OR) of mortality among kidney transplant recipients with AF was 1.86 (3 studies; 95% CI: 1.03–3.35). In addition, AF is also associated with death-censored allograft loss (2 studies; OR: 1.55, 95% CI: 1.02–2.35) and stroke (3 studies; OR: 2.54, 95% CI: 1.11–5.78) among kidney transplant recipients. Despite advances in medicine, incidence of AF following kidney transplant does not seem to decrease over time. In addition, there is a significant association of AF with increased mortality, allograft loss, and stroke after kidney transplantation.

## 1. Introduction

Atrial fibrillation (AF) is one of the most frequent diagnoses, affecting 3 to 6 million people in the United States, and almost 30 million people worldwide [1,2,3,4]. Global prevalence of AF has continued to rise and is expected to reach 50 million people by 2050 [1,2,3,4,5,6]. Patients with AF carry a higher risk of mortality and adverse cardiovascular events including stroke [7,8]. Among end-stage renal disease (ESRD) patients, given hypercoagulable state [9,10] and hemodynamic changes during dialysis [11], the prevalence of AF is exceptionally high, approximately 12% [12,13], when compared to the prevalence in the general patient population of 2.5% [14]. One-year mortality risk of ESRD patients with AF is twice higher than those without AF [12,15].

Kidney transplantation is the treatment of choice for ESRD and improves the survival and quality of life for the majority of ESRD patients when compared to dialysis [16,17,18,19,20]. While advances in immunosuppression and surgical techniques have led to significant improvement in short-term survival of the renal allograft [21], long-term renal allograft survival is still an ongoing concern [22,23]. While reduced kidney function is an important risk factor for AF development [24], the improvement of renal function after successful kidney transplantation may affect the incidence of AF and potential consequences of AF [25,26,27,28,29,30,31,32]. Conversely, immunosuppressive agents, insulin resistance, and metabolic syndrome after kidney transplantation may also impact on the potential consequences of AF [29,33,34,35]. In spite of progress in transplant medicine, the trends of incidence of AF following kidney transplantation over time remain unclear [5,25,26,27,28,29,30,31,32,36,37].

Thus, this meta-analysis was conducted with the aim to summarize all available data on (1) prevalence of pre-existing AF and/or incidence of AF following kidney transplantation; (2) the outcomes of kidney transplant recipients with AF; and (3) the trends of estimated incidence of AF following kidney transplantation over time.

## 2. Methods

### 2.1. Search Strategy and Literature Review

The protocol for this meta-analysis is registered with PROSPERO (International Prospective Register of Systematic Reviews; no. CRD42018086192). A systematic literature search of MEDLINE (1946 to March 2018), EMBASE (1988 to March 2018), and the Cochrane Database of Systematic Reviews (database inception to March 2018) was conducted (1) to assess prevalence of pre-existing AF and/or incidence of AF following kidney transplantation and (2) to evaluate the outcomes of kidney transplant recipients with AF. The systematic literature review was undertaken independently by two investigators (C.T. and R.C.) using the search strategy that combined the terms of “kidney” or “renal” AND “transplant” OR “transplantation” AND “atrial fibrillation”, which is provided in Supplementary materials. No language limitation was applied. A manual search for conceivably relevant studies using references of the included articles was also performed. This study was conducted by the STROBE (Strengthening the Reporting of Observational Studies in Epidemiology) [38] and the PRISMA (Preferred Reporting Items for Systematic Reviews and Meta-Analysis) statement [39].

### 2.2. Selection Criteria

Eligible studies must be clinical trials or observational studies (cohort, case-control, or cross-sectional studies) that reported prevalence of pre-existing AF or incidence of AF following kidney transplantation or outcomes of kidney transplant recipients with AF. They must provide the data on prevalence or incidence or effect estimates relative risks (RR), odds ratios (OR), or hazard ratios (HR) with 95% confidence intervals (CI). Retrieved articles were individually reviewed for eligibility by the two investigators (C.T. and R.C.). Discrepancies were addressed and solved by a third investigator (W.C.) and joint consensus. Inclusion was not limited by the size of study. The Newcastle-Ottawa quality assessment scale was applied to evaluate the quality of study for case-control study and outcome of interest for cohort study [40], as shown in Table 1.

### 2.3. Data Abstraction

A structured data collecting form was used to obtain the following information from each study including title, name of the first author, publication year, year of the study, country where the study was conducted, demographic data of kidney transplant patients, methods used to identify AF, prevalence of pre-existing AF, incidence of postoperative AF, patient outcomes following kidney transplantation, adjusted effect estimates with 95% CI and covariates that were adjusted for in the multivariable analysis.

### 2.4. Statistical Analysis

Analyses were performed utilizing the Comprehensive Meta-Analysis 3.3 software (version 3; Biostat Inc, Englewood, NJ, USA). Adjusted point estimates from each study were consolidated by the generic inverse variance approach of DerSimonian and Laird, which designated the weight of each study based on its variance [41]. Given the possibility of between-study variance, we used a random-effect model rather than a fixed-effect model. Cochran’s Q test and *I*^2^ statistic were applied to determine the between-study heterogeneity. A value of *I*^2^ of 0% to 25% represents insignificant heterogeneity, 26% to 50% low heterogeneity, 51% to 75% moderate heterogeneity and 76–100% high heterogeneity [42]. The presence of publication bias was assessed by the Egger test [43].

## 3. Results

**Figure 1:**
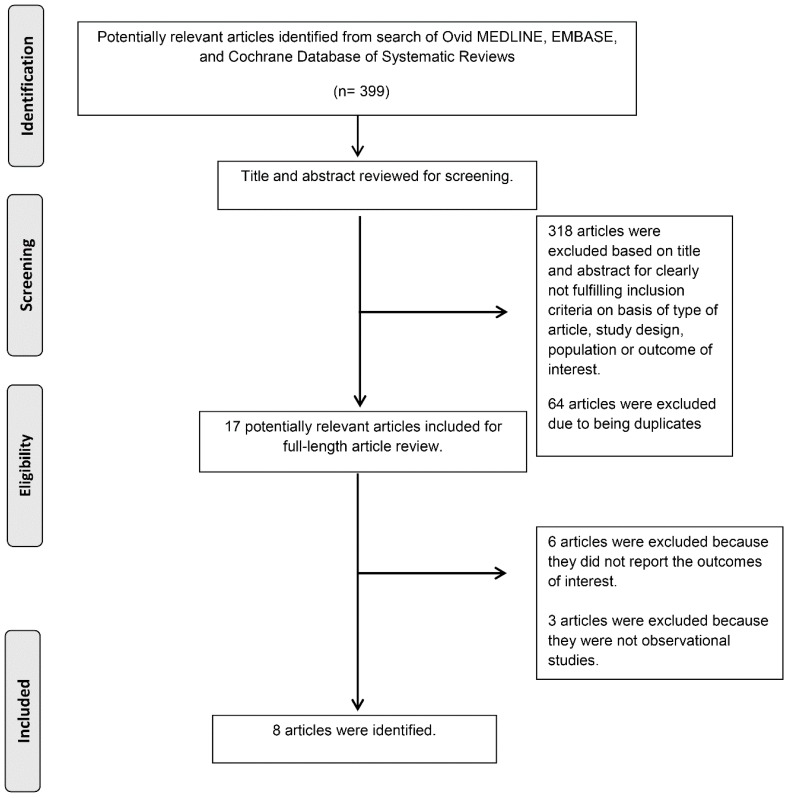
Outline of our search methodology.

A total of 399 potentially eligible articles were identified using our search strategy. After the exclusion of 382 articles based on title and abstract for clearly not fulfilling inclusion criteria on the basis of type of article, study design, population, or outcome of interest, and due to some being duplicates, 17 articles were left for full-length review. Six of them were excluded from the full-length review as they did not report the outcome of interest while three articles were excluded because they were not observational studies. Thus, we included 8 cohort studies [25,26,27,28,29,30,31,32] into the final analysis with 137,709 kidney transplant recipients that were enrolled. Kappa coefficient of agreement for the investigators was 0.87. Disagreements were resolved by a third researcher (W.C.) and joint consensus. The literature retrieval, review, and selection process are demonstrated in Figure 1. The characteristics and quality assessment of the included studies are presented in Table 1 [25,26,27,28,29,30,31,32].

### 3.1. Prevalence of Pre-Existing AF and Incidence of AF after Kidney Transplantation

**Figure 2:**
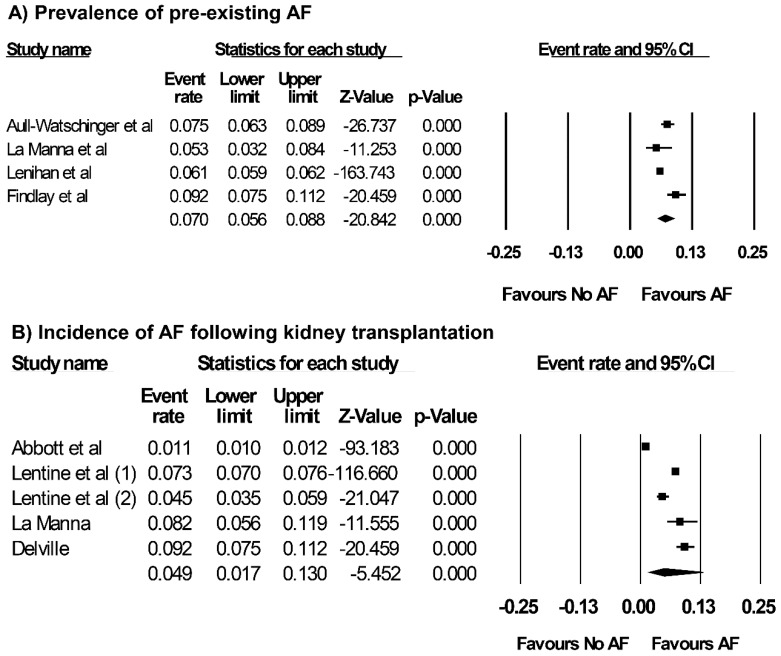
Forest plots of the included studies [5,25,26,28,29,30,31,32] assessing (**A**) prevalence of pre-existing AF in patients undergoing kidney transplantation, and (**B**) incidence of AF following kidney transplantation. A diamond data marker represents the overall rate from each included study (square data marker) and 95% confidence interval.

Overall, the pooled estimated prevalence of pre-existing AF in patients undergoing kidney transplantation was 7.0% (95% CI: 5.6–8.8%, *I*^2^ = 86%, Figure 2) and the pooled estimated incidence of AF following kidney transplantation was 4.9% (95% CI: 1.7–13.0%, *I*^2^ = 99%, Figure 2). When the data were limited only to new-onset AF after kidney transplant recipients, pooled estimated incidence of new-onset AF was 4.2% (95% CI: 1.6–10.6%, *I*^2^ = 94%).

**Figure 3:**
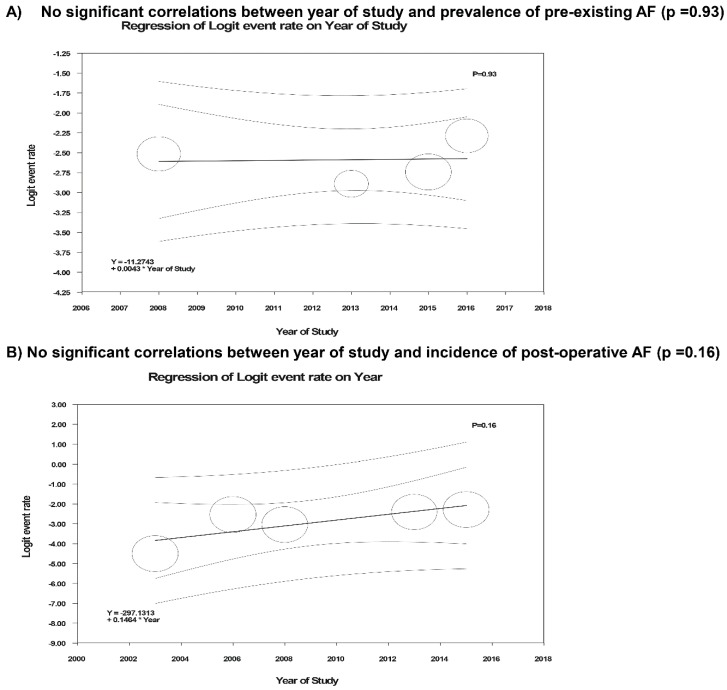
Meta-regression analyses showed (**A**) no significant correlations between year of study and either prevalence of pre-existing AF (*p* = 0.93) or (**B**) post-operative AF after kidney transplantation (*p* = 0.16). The solid black line represents the weighted regression line based on variance-weighted least squares. The inner and outer broken lines show the 95% confidence interval and prediction interval around the regression line. The circles indicate log event rates in each study.

Meta-regression analyses were performed and showed no significant correlations between year of study and either prevalence of pre-existing AF (*p* = 0.93) or post-operative AF after kidney transplantation (*p* = 0.16), as shown in Figure 3.

### 3.2. Risk Factors of AF and Outcomes of Kidney Transplant Recipients with AF

**Figure 4:**
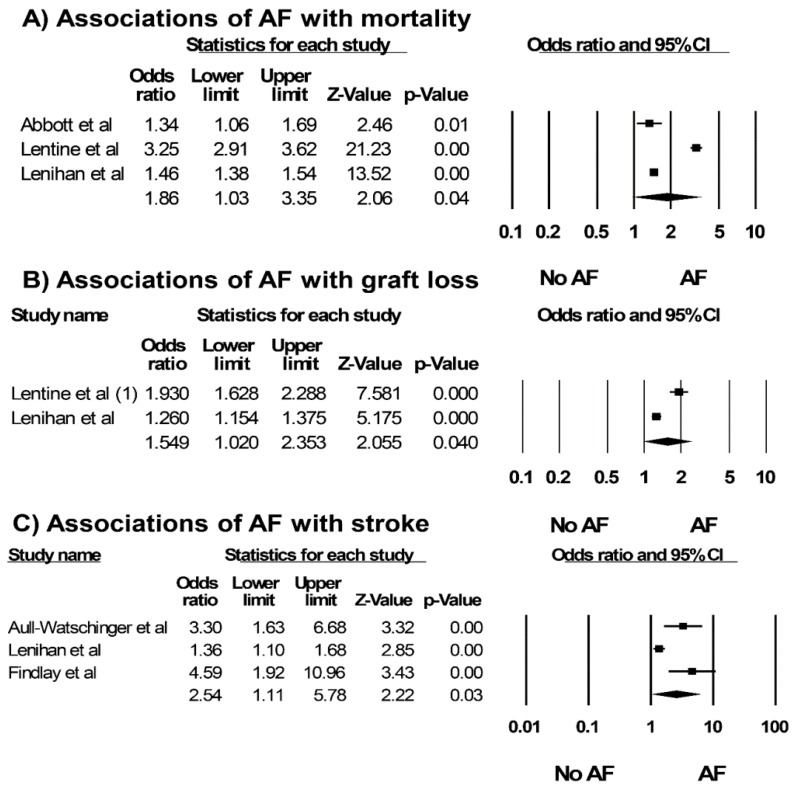
Associations of AF with (**A**) mortality, (**B**) death-censored allograft loss and (**C**) stroke among kidney transplant recipients from included studies [5,26,28,29,30,31]. A diamond data marker represents the overall rate from each included study (square data marker) and 95% confidence interval.

Reported risk factors associated with AF after kidney transplantation are demonstrated in Table 2 [25,29,30,31]. Older recipient age [25,29,30], higher BMI, and history of coronary artery disease/acute myocardial infarction have been demonstrated as important risk factors for AF after kidney transplantation. The pooled OR of mortality among kidney transplant recipients with AF was 1.86 (3 studies; 95% CI: 1.03–3.35, *I*^2^ = 98%, Figure 4). In addition, AF is associated with death-censored allograft loss (2 studies; OR: 1.55, 95% CI: 1.02–2.35, *I*^2^ = 94%, Figure 4) and stroke (3 studies; OR: 2.54, 95% CI: 1.11–5.78, *I*^2^ = 83%, Figure 4) among kidney transplant recipients.

### 3.3. Evaluation for Publication Bias

Funnel plots (Supplementary Figures S1 and S2) and Egger’s regression asymmetry tests were performed to evaluate for publication bias in analyses evaluating prevalence of pre-existing AF and incidence of postoperative AF in kidney transplant patients, respectively. There was no significant publication bias in both analyses evaluating prevalence of pre-existing AF and incidence of postoperative AF in kidney transplant patients, *p* = 0.33 and *p* = 0.68, respectively. 

## 4. Discussion

In this meta-analysis, we demonstrated that ESRD patients who underwent kidney transplantation had a prevalence of AF of 7.0%. In addition, our study showed the pooled incidence of AF after kidney transplantation of 4.9%. Our findings showed a statistically significant association of AF after kidney transplantation with 1.9-fold increased risk of mortality, 1.6-fold increased risk of renal allograft loss, and 2.5-fold increased risk of stroke after kidney transplantation.

Based on the findings from our meta-analysis, the prevalence of pre-existing of AF among ESRD patients undergoing kidney transplantation is higher than the 2.5% prevalence in the general patient population of, [14] although it is lower than the 12% prevalence in overall ESRD patients [12,13], and the 6% prevalence in patients with end-stage liver disease undergoing liver transplantation [44]. Since not all ESRD patients are candidates for kidney transplantation due to their significant comorbidities, it is not surprising that the prevalence of AF among ESRD patients undergoing kidney transplantation from our study is lower than the prevalence among ESRD population in general. Following kidney transplantation, we demonstrated that approximately 4% of kidney transplant recipients developed new-onset AF. There are several explanations as to why kidney transplantation promotes the occurrence of AF during postoperative period. First, although hemodynamic instability during kidney transplantation is not as common as liver transplantation [45,46], conventional postoperative stress could provoke AF through hemodynamic instability [47,48,49]. In addition, hypertension and obesity, known risk factors for AF, are also common among kidney transplant recipients [31,50]. Furthermore, immunosuppressive agents are known to be associated with insulin resistance and metabolic syndrome after kidney transplantation (such as calcineurin inhibitors-induced diabetes mellitus [33] and mammalian target of rapamycin (mTOR) inhibitors-associated dyslipidemia [34]), which are important risk factors for AF [29,35]. Consistently, the majority of the included studies in our systematic reviews identified older recipient age [25,29,30], higher BMI, and a history of coronary artery disease/acute myocardial as predictors for AF development after kidney transplantation.

Leading causes of long-term mortality in kidney transplant recipients are cardiovascular complications, which, other than AF, include heart failure and myocardial infarction [51,52,53]. These cardiovascular complications were also considered as potential risk modification strategy that should not be overlooked. In general population, AF can put the patients at higher mortality risk compared to those without AF [54]. In addition to mortality risk, our study also revealed the associations of AF with renal allograft loss and stroke among kidney transplant recipients. There are several mechanisms that put the kidney transplant patients with AF at higher risk of postoperative morbidity and mortality compared to those without AF. Although high mortality in kidney transplant recipients with AF may have been contributed by other cardiovascular risks associated with AF (such as congestive heart failure and coronary artery disease) at the time even before kidney transplantation, studies have also demonstrated that AF after kidney transplantation itself is independently associated with increased mortality, morbidity, number of hospitalizations, and high healthcare cost [26,27,29,30]. In addition, amiodarone-tacrolimus interaction leading to QT prolongation and fatal arrhythmias in kidney transplantations have been reported [55,56]. Thus, this combination should be cautiously used with careful monitoring.

There are several limitations in our systematic review and meta-analysis. First, statistical heterogeneities were present in our study. Possible explanations for this heterogeneity include the differences in the methodology of diagnosis of AF and patient characteristics in each study. Despite these heterogeneities, meta-regression demonstrated no significant correlation between year of study and incidence of AF after kidney transplantation, representing no potential improvement in incidence of AF after kidney transplantation over time. Second, duration of follow up during the postoperative period by several studies assessing AF was just until hospital discharge or up to one-year post-transplantation [25,32]. Although the majority of cases of AF following kidney transplantation developed within the first year after kidney transplantation [25,26,27,28,29,30,31,32], the true incidence of AF might have been slightly higher. Third, while we demonstrated high mortality and stroke risks in kidney transplant recipients with AF, it remains unclear if anticoagulation use (warfarin and novel agents) in kidney transplant patients would improve patient outcomes; future clinical trials of anticoagulation use in the kidney transplant population with AF are needed [27,28,57]. Last, this is a meta-analysis of observational studies, and as such, it could only reveal association, not a causal-effect relationship, between kidney transplantation and AF.

## 5. Conclusions

In spite of progress in transplant medicine, incidence of AF following kidney transplants does not seem to decrease over time. When compared to those without AF, this meta-analysis shows that kidney transplant recipients with AF may carry higher risks of mortality, renal allograft loss, and stroke.

## Figures and Tables

**Table:** (**a**).

Study	Aull-Watschinger et al. [26]	La Manna et al. [25]	Lenihan et al. [5]	Findlay et al. [28]
Country	Austria	Italy	USA	UK
Study design	Cohort	Cohort	Cohort	Cohort
Study year	2008	2013	2015	2016
Total number	1633	304	62706	956
Patients	Kidney or kidney-pancreas transplant patients in a single center	Kidney or kidney/liver transplant patients in a single center	Kidney transplant patients in the US renal Data System	Functioning kidney transplant patients in a single hospital
Living donor	174/1633 (11%)	N/A	10409/62706 (17%)	N/A
Anticoagulation	Antiplatelet or anticoagulation 454/1633 (28%)	N/A	N/A	Warfarin 137/956 (14%)
AF ascertainment	History of AF before kidney transplant; identified by medical record review	Postoperative AF until hospital discharge; identified by medical record review	History of AF before kidney transplant; identified by identified by ICD-9 code 427.3x in Medicare claims	History of AF before kidney transplant; identified by medical record review
Pre-operative AF	122/1633 (7.5%)	16/304 (5.3%)	3794/62706 (6.1%)	88/956 (9.2%)
Estimated prevalence				
Post-operative AF	N/A	POAF 25/304 (8.2%)	N/A	N/A
Estimated prevalence		De novo POAF 21/304 (6.9%)		
Follow-up	Median 4 (IQR 1.5–6.7) years	Until hospital discharge	Mean 4.9 years	Median 5.4 years
Outcomes	TIA/stroke 3.30 (1.63–6.67)	POAF and myocardial ischemia 11.58 (0.70–191.06)	Death 1.46 (1.38–1.54)	Stroke 4.59 (1.92–10.94)
			All-cause graft loss 1.41 (1.34–1.48)	Ischemic stroke in AF 1.72% at 1 year and 4.07% at 3 years
			Death-censored graft loss 1.26 (1.15–1.37)	
			Death-censored ischemic stroke 1.36 (1.10–1.68)	Ischemic stroke risk in non-AF 0.72% at 1 year and 2.07% at 3 years
Confounder adjustment	DM, ejection fraction, C-reactive protein, hyperlipidemia, polycystic kidney disease, duration of dialysis, sex, age, degree of carotid stenosis	None	Age, sex, race, BMI, cause of ESRD, dialysis vintage and modality, SNF utilization, number of hospital days and non-nephrology clinic visits, previous transplants, comorbidities, blood type, PRA, donor age and sex, transplant type, HLA mismatches, cold ischemia time	None
Newcastle-Ottawa Scale	S 3	S 3	S 4	S 3
	C 2	C 2	C 2	C 2
	O 3	O 3	O 3	O 3

AF, Atrial Fibrillation; BMI, body mass index; DM, diabetes mellitus; ESRD, end-stage renal disease; HLA, human leukocyte antigen; ICD-9, international classification of diseases, ninth; IQR, interquartile range; N/A, not available; POAF, postoperative atrial fibrillation; PRA, panel reactive antibody; S, C, O, selection, comparability, and outcome; SNF, skilled nursing facility; TIA, transient ischemic attack.

**Table:** (**b**).

Study	Abbott et al. [29]	Lentine et al. [30]	Lentine et al. [31]	Delville et al. [32]
Country	USA	USA	USA	France
Study design	Cohort	Cohort	Cohort	Cohort
Study year	2003	2006	2008	2015
Total number	39628	31136	1102	244
Patients	Kidney transplant patients in the US Renal Data System	Kidney transplant patients in the US Renal Data System	Kidney transplant patients in a single center	Kidney transplant patients aged >50 years in a single center
Living donor	12259/39628 (31%)	6993/31136 (22%)	344/1102 (31%)	N/A
Anticoagulation	N/A	N/A	N/A	N/A
AF ascertainment	Hospitalizations for a primary diagnosis of AF; identified by ICD-9 code 427.31	AF after kidney transplant; identified by ICD-9 code 427.3x	New-onset atrial fibrillation after kidney transplant; identified by ECG	New-onset atrial fibrillation after kidney transplant; identified by medical record review and ECG
Pre-operative AF	N/A	N/A	N/A	N/A
Estimated prevalence				
Post-operative AF	432/39628 (1.1%)	New-onset AF	5-year 50/1102 (4.5%)	13/244 (5.3%)
		At 6 months 810/31136 (2.6%)		
		At 12 months 1121/31136 (3.6%)		
Estimated prevalence				
		At 36 months 2273/31136 (7.3%)		
Follow-up	Mean 1.89 ± 1.15 years	Up to 36 months	5 year	1 year
Outcomes	Mortality 1.34 (1.06–1.69)	Mortality 3.25 (2.92–3.63)	N/A	N/A
		Death-censored graft loss 1.93 (1.63–2.29)		
		All-cause graft loss 2.88 (2.60–3.12)		
Confounder adjustment	Adjusted but not specified	Age, sex, race, education, employment, BMI, causes of ESRD, dialysis duration, sensitization, comorbid conditions, smoking, alcohol abuse. donor age and source, donor CMV status, degree of HLA matching, induction and maintenance immunosuppression, DGF, post-transplantation complications	N/A	N/A
Newcastle-Ottawa Scale	S 4	S 4	S 3	S 3
	C 1	C 2	C 2	C 2
	O 3	O 3	O 3	O 3

AF, Atrial Fibrillation; CMV, Cytomegalovirus; DGF, delayed graft function; ECG, electrocardiogram; HLA, human leukocyte antigen; ICD-9, international classification of diseases, ninth; N/A, not available; SNF, skilled nursing facility; S, C, O, selection, comparability, and outcome.

**Table 2:** Risk factor associated with AF after kidney transplantation.

Studies	Follow-up Time	Risk Factor Associated with AF after Kidney Transplantation
Abbott et al. [29]	Mean 1.89 ± 1.15 years	Older recipient age, higher BMI, DGF, rejection, ESRD due to hypertension, cyclosporine use, Graft loss
Lentine et al. [30]	Up to 36 months	Older recipient age, male sex, Caucasian, non-Hispanic, ESRD due to hypertension, longer dialysis duration before transplant, CAD, DGF, older donor age, post-transplantation complications (hypertension, anemia, new-onset diabetes, MI, graft failure)
La Manna et al. [25]	Until hospital discharge	Older age, kidney/liver transplant, history of acute myocardial infarction
Lentine et al. [31]	5 year	BMI

AF, Atrial Fibrillation; BMI, body mass index; CAD, coronary artery disease; DGF, delayed graft function; ESRD, end-stage renal disease; MI, myocardial infarction.

## Supplementary Materials

The following are available online at http://www.mdpi.com/2077-0383/7/10/370/s1, Figure S1: Funnel plot evaluating prevalence of pre-existing AF in kidney transplant patients, Figure S2: Funnel plot evaluating incidence of postoperative AF in kidney transplant patients, online Data S1: Search terms for systematic review.
