# Supplementary material for: Epidemiology and Prognostic Importance of Atrial Fibrillation in Kidney Transplant Recipients: A Meta-Analysis
Source: J Clin Med. 2018 Oct 19;7(10):370. doi: 10.3390/jcm7100370 (PMC6210475; doi:10.3390/jcm7100370)

Online supplementary data 1

Search terms for systematic review.

Databases: Ovid MEDLINE

1. exp atrial fibrillation/
2. atrial fibrillation$.mp
3. 1 or 2
4. exp transplantation/
5. transplant$.mp
6. 4 or 5
7. kidney.mp
8. renal.mp
9. 7 or 8
10. 6 and 9
11. 3 and 10

Databases: EMBASE:

All fields: 'atrial fibrillation'

AND All fields: 'kidney transplantation'

Database: Cochrane Databases

Search all text '"atrial fibrillation" AND "kidney transplantation"

Supplementary Figure 1: Funnel plot evaluating prevalence of pre-existing AF in kidney transplant patients,

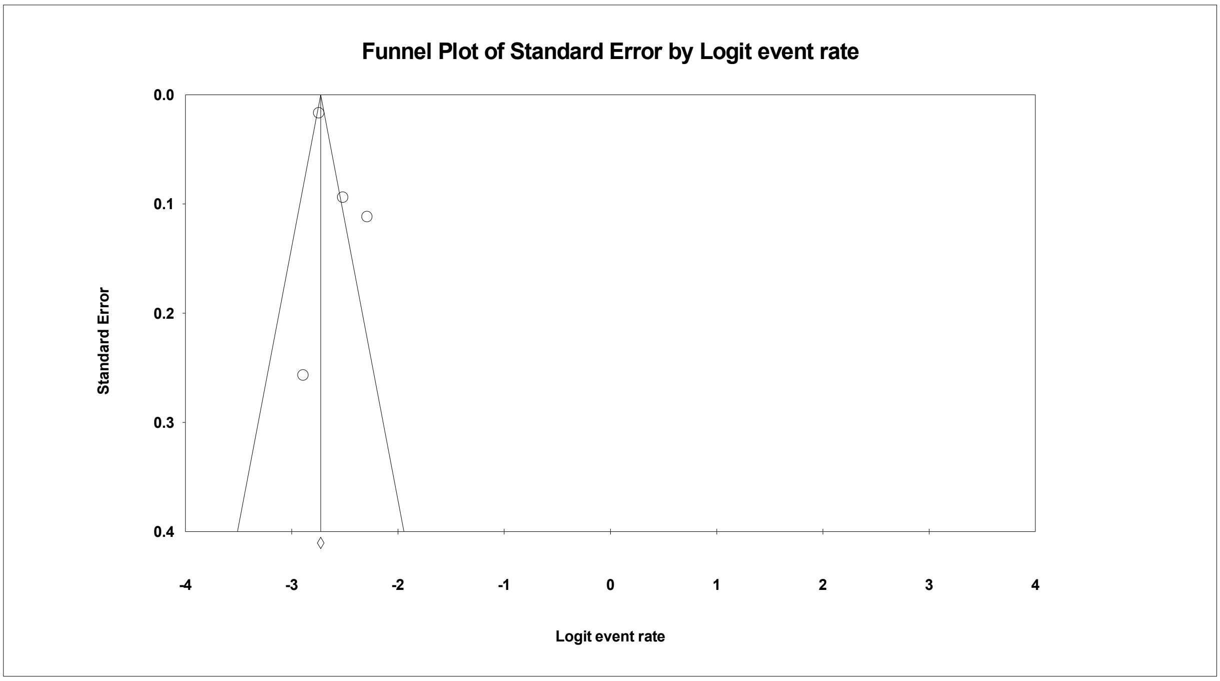

Supplementary Figure 2: Funnel plot evaluating incidence of postoperative AF in kidney transplant patients

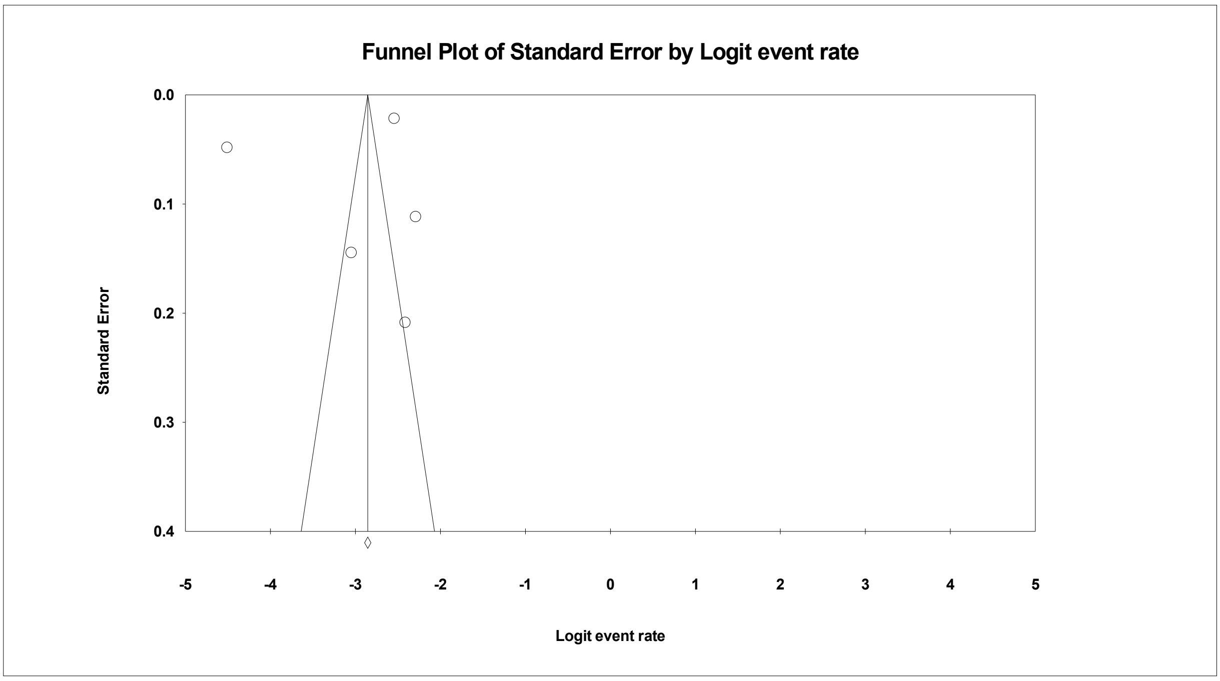

Supplement: Supplementary file 1 [file jcm-07-00370-s001.pdf]
